# Combination of Stable Isotope Analysis and Chemometrics to Discriminate Geoclimatically and Temporally the Virgin Olive Oils from Three Mediterranean Countries

**DOI:** 10.3390/foods9121855

**Published:** 2020-12-12

**Authors:** Nicasio T. Jiménez-Morillo, Vera Palma, Raquel Garcia, Cristina Barrocas Dias, Maria João Cabrita

**Affiliations:** 1 MED—Mediterranean Institute for Agriculture, Environment and Development, Universidade de Évora, Pólo da Mitra, Ap. 94, 7006-554 Évora, Portugal; ntjm@uevora.pt (N.T.J.-M.); raquelg@uevora.pt (R.G.); 2 HERCULES, Universidade de Évora, Palácio do Vimioso, 7000-089 Évora, Portugal; vscpalma@uevora.pt (V.P.); cmbd@uevora.pt (C.B.D.); 3 Departamento de Fitotecnia, Escola de Ciências e Tecnologia, Universidade de Évora, Núcleo da Mitra, Ap. 94, 7006-554 Évora, Portugal; 4 Departamento de Química, Escola de Ciências e Tecnologia, Universidade de Évora, Rua Romão Ramalho, 59, 7000-671 Évora, Portugal

**Keywords:** olive oil, meteoric water line, EA-IRMS, stable isotopes

## Abstract

The knowledge of the isotopic composition of virgin olive oil (VOO) allows the evaluation of authenticity and geographical origin, being an important tool against fraud. This study aimed to assess if VOOs produced in three Mediterranean regions could be discriminated on the basis of multivariate statistical analysis of geoclimatic and isotopic data. A total of 138 geo-referenced VOO samples from Portugal, France and Turkey from two different cultivation years were collected. The isotopic composition (*δ*^13^C, *δ*^2^H and *δ*^18^O) of VOOs was obtained using an elemental analyzer coupled to an isotope ratio mass spectrometer (EA-IRMS). One-way analysis of variance for *δ*^13^C, *δ*^2^H and *δ*^18^O showed some significant differences either between crop years or geoclimatic conditions. Based on multiple regression analyses using meteorological and geographical parameters, a meteoric water line for olive oil from Portugal, France and Turkey, in two harvest years, were created to assess the impact of climate change on their *δ*^2^H and *δ*^18^O values. Principal component analysis and Linear Discriminant Analysis, used to sort samples according to geoclimatic origin, performed best for French and Portuguese olive oils. In light of the results, multivariate isotopic analysis of VOO samples may discriminate not only between geoclimatic regions but also among cultivation years.

## 1. Introduction

Traceability of olive oils is still a popular research topic, addressed in many different ways, including exploring the benefits of different analytical tools due to the increased demand for an effective analytical methodology that can verify the geographic origin of olive oils. As claim of origin is becoming more important, so is the need for new tools to establish it. Traditional chromatography techniques (gas or liquid) coupled to mass spectrometry [1,2,3] or UV detection [4], or the “more environmentally friendly” analytical methods based on spectroscopy [5,6] have been used for this purpose.

However, olive oil stable isotope ratio analysis (SIRA), either in bulk, using Isotope Ratio Mass Spectrometry (IRMS) coupled to an elemental analyzer (EA), or in a class of compounds using IRMS coupled with a gas chromatographer (GC), is nowadays used more often to achieve separation among olive oils from different geographical origins [7,8,9,10,11,12]. The SIRA is based on the fact that the stable isotope content of bio-elements reflects the geo-climatic characteristics of the area of production [13] and was initially performed in bulk analysis. In the case of olive oil, bulk δ^13^C, δ^18^O and δ^2^H determinations allowed the differentiation among different geographic origins, at least in some cases. With innovations in this analytical technique that now allow the study of stale isotopes in specific compounds, like fatty acids, or volatile compounds, more detailed information can be retrieved allowing improvement in geographical discrimination.

Oxygen and hydrogen isotope ratios (δ^18^O and δ^2^H), as a natural tracer, have been effectively used in diagnosing water sources [14], as well as a marker of authenticity and origin of food or beverages. Particularly, these isotopes have been employed to trace the authenticity and geographical origin of European extra-virgin olive oils [12,15]. The stable isotope compositions of oxygen and hydrogen in soil and ground waters are controlled by local precipitation in the recharge and catchment areas, depending on various geoclimatic factors (e.g., temperature, distance from the sea, amount of atmospheric precipitation, etc.) [16,17]. Therefore, the variations in δ^2^H and δ^18^O values in plants and their products, through their relationship with humidity-precipitation and temperature conditions during plant growth, may allow the tracing of the geographic origin of vegetable oils [18,19,20,21].

Bearing this in mind, our study aimed to assess if VOOs from three different Mediterranean countries (Portugal, France and Turkey) could be discriminated on the basis of multivariate statistical analysis of geoclimatic and isotopic data (δ^13^C, δ^2^H and δ^18^O) obtained from bulk analysis of 138 varietal VOOs.

## 2. Materials and Methods

### 2.1. Samples and Experimental Design

A total of 138 varietal VOO (*Olea europaea* L.) samples from three different Mediterranean countries, Portugal (27 in 2016 and 39 in 2017), France (25 in 2016 and 25 in 2017) and Turkey (10 in 2016 and 11 in 2017), were collected from November to December in 2016 and in 2017. For the majority of the selected VOO samples, approximately 5 kg of each different olive variety was processed separately in an Abencor^®^ system (Seville, Spain) within 24 h after harvesting. Fruits were crushed with a hammer mill and the olive paste was malaxed at 25 °C for 30 min in an olive paste mixer. Finally, the olive oil was separated by centrifugation. Other samples were taken directly from olive oil mills to avoid possible undeclared mixtures with olive oils from other cultivars and geographical origins, before bottling. They were stored in dark-brown glass bottles at 20 °C in the dark. The VOO samples were perfectly geo-referenced, obtaining data for latitude (UTM), longitude (UTM), altitude (m.a.s.l.), sea distance (km), mean annual temperature (°C) and mean annual rainfall (mm) (Table S1).

### 2.2. Stable Isotope Analysis

The carbon, hydrogen and oxygen isotope composition ratios (^13^C/^12^C, ^2^H/^1^H and ^18^O/^16^O, respectively) of VOO samples were determined by elemental analysis/isotope ratio mass spectrometry (EA/IRMS) following the procedure described by Jiménez-Morillo et al. [12]. Briefly, bulk isotopic signature of light elements (δ^13^C, δ^2^H and δ^18^O) was analysed using a Flash 2000 HT with two reactors (combustion and pyrolysis, respectively). The elemental analyzer is coupled by a ConFlo IV interface unit to a continuous flow Delta V Advantage isotope ratio mass spectrometer (IRMS) (Thermo Scientific, Bremen, Germany). Isotopic ratios “δ” are reported in milli Urey (mUr) from appropriate standards recognized by the International Atomic Energy Agency (IAEA). The standard deviation of bulk δ^13^C, δ^2^H and δ^18^O were ± 0.1, 1.0 and 0.5 mUr, respectively. Each sample was measured in duplicate (*n* = 2).

### 2.3. Statistical Analysis

Multivariate statistical analysis of data was carried out with Statgraphics Centurion XV software (Statgraphics Technologies, Inc, The Plains, VA, USA). Principal component analysis (PCA) was used for simultaneous ordination of different geographic and climatic variables and the δ^13^C, δ^2^H and δ^18^O values, illustrating their mutual relationships. Multiple linear regression (MLR) was applied to generate an isotopic forecasting model by harvest year, using geographic and climatic factors as independent variables. The validation of the model was confirmed by the production of spurious models computed from the fully randomized dependent variables (isotopic composition of hydrogen, oxygen and carbon) from the 138 VOO samples from three different Mediterranean countries in two harvest years. The alternative models displayed a poor correlation (*p* > 0.05) with geoclimatic variables (data not shown). Therefore, the well-correlated models generated with experimental data were not overfitted [12,22]. Linear discriminant analysis was also carried out to evaluate whether olive oil samples from the three countries could be easily separated based on isotopic data. The statistical significance of each discriminant function was evaluated by Wilk’s lambda [23] and the predictive ability of the LDA model was evaluated by leave-one-out cross validation.

## 3. Results and Discussion

### 3.1. Stable Isotope Analysis of VOO Samples

**Figure 1:**
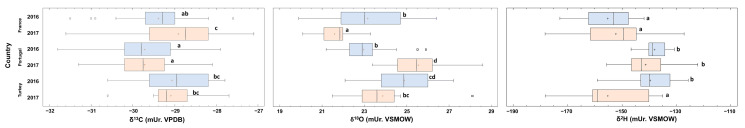
Boxplots of the bulk carbon (*δ*^13^C values, **a**), oxygen (*δ*^18^O values, **b**) and hydrogen (*δ*^2^H values, **c**) isotope composition of olive oils from three Mediterranean countries (France, Portugal and Turkey) in two harvest years (2016 and 2017). Boxplots display the ranges, lower and upper quartiles (Q1, Q3), and the median (Q2). Box plots with different letters indicate significant difference (ANOVA; Means compared using Tukey test, *p* = 0.05).

Table S1 collects the carbon, hydrogen and oxygen isotope values of VOO samples from production areas in France, Portugal and Turkey in two harvest years. Concerning δ^13^C value, the Portuguese VOO samples ranged between −31.8 and −27.9 mUr for 2016, and between −31.3 and −28.1 mUr for 2017. In the case of French samples, the δ^13^C composition ranged between −31.5 and −27.6 mUr for 2016, and between −31.6 and −27.1 mUr for 2017. Finally, the VOO samples from Turkey showed δ^13^C value ranging between −30.6 and −27.8 mUr, and −30.6 and −27.7 mUr for 2016 and 2017, respectively. These isotopic values are in accordance with those reported by Gumus et al. [9] for olive oils from western Turkey. The δ^13^C values of the VOO samples from the three Mediterranean countries were within the typical value of C_3_ photosystem plants [24]. However, as observed, there were significant differences between location and harvest years. One-way analysis of variance (one-way ANOVA) using δ^13^C composition (Figure 1a) as independent variable (IV) indicated that Portuguese and Turkish VOOs are significantly (*p* < 0.05) different between themselves, but not between respective cultivation years. The French samples were significantly different (*p* < 0.05) between cultivation years. Traditionally, it has been accepted that δ^13^C composition of food-stuff depends on the localization of the production area, i.e., on the geomorphological and climatic conditions, such as temperature, rainfall, sea distance, longitude and latitude [12]. These factors influence leaf stomata opening and hence the photosynthesis yield [25], as dry and warm conditions may produce a restriction in the opening time of stomas, limiting the admission of atmospheric CO_2_ to the leaf, causing an increase in δ^13^C value. The existence of no significant differences between harvest year in Portugal and Turkey may be related to the similitude in mean annual temperature between 2016 and 2017 (Table S2). However, in French regions a significant difference between harvest year was observed, which was also displayed in the temperature values (Table S2).

On the other hand, hydrogen and oxygen isotope composition of VOO samples (Table S1) showed particular trends for each one. In the case of δ^18^O, the values of EVOOs from Portugal ranged between 21.2 and 25.9 mUr for 2016 and between 23.4 and 28.6 mUr for 2017, while from France the value ranged between 19.9 and 26.4 mUr for 2016 and between 20.1 and 23.3 mUr for 2017. For Turkish VOO samples, the δ^18^O composition was between 22.1 and 27.2 mUr for 2016, and between 21.5 and 28.1 mUr for 2017. The statistical analysis of δ^18^O values (Figure 1b) displayed significant (*p* < 0.05) differences amongst cultivation years for Portuguese and French samples. In addition, the VOOs cultivated in 2017 were considerably different between the three analyzed Mediterranean countries, while for 2016 only Turkish VOOs were significantly different. With respect to δ^2^H composition in 2016 and 2017 (Table S1), the values ranged: (i) between −146.6 and −127.1 mUr, and between −155.6 and −122.0 mUr for Portugal; (ii) between −186.7 and −141.8 mUr, and between −178.2 and −127.1 mUr for France; and (iii) between −159.0 and −125.6 mUr, and between −178.1 and −135.1 mUr for Turkey. One-way ANOVA analysis of δ^2^H values (Figure 1c) showed that only the samples from Turkey were significantly (*p* < 0.05) different according to the cultivation year. In contrast, the samples from Portugal and France are significantly (*p* < 0.05) different from each other, which may be directly related to a quite different climate, mainly temperature and precipitation (Table S2). With respect to Turkey, the significant differences between harvest years may be due to specific climatological events that could have altered the isotopic composition of hydrogen. This hypothesis may be supported by the fact that the δ^2^H and δ^18^O composition of foodstuffs are significantly correlated to (1) the local climatic conditions (temperature and precipitation) [12,16,26]; (2) soil and plant evapotranspiration [27,28]; (3) air humidity; and (4) fractionation associated with the biosynthesis of plant tissues [29,30]. Therefore, the differences observed between countries and harvest years may be related to the above mentioned factors. However, the existence of irrigation activities may also modify the isotopic composition of hydrogen and oxygen [31,32].

### 3.2. Meteoric Water Line

**Figure 2:**
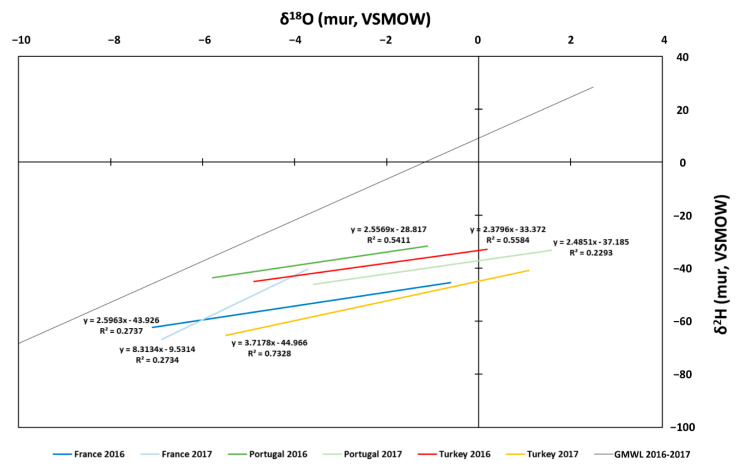
Local Meteoric Water Lines (LMWL) generated from the bulk *δ*^18^O and *δ*^2^H of virgin olive oil (VOO) samples. In all cases, the correlation value was highly significate (*p* < 0.05). The global meteoric water line (GMWL) is shown for reference.

Figure 2 shows the correlation between δ^2^H and δ^18^O of VOO samples from the three Mediterranean countries in two harvest years. In agreement with other researchers, the H and O isotope values of VOO samples are directly correlated to the meteoric water uptake by olive plants, so it has been observed that the H isotope value of olive oil is about 100 times lower than the value of rainwater, while the O isotope value is about 27 times higher than that of rainwater [30,33]. Therefore, in this work, these criteria were used to generate the local meteoric water line (LMWL) for each location and harvest year (Figure 2). In addition, the correlation lines were compared with the global meteoric water line (GMWL). The GMWL was generated using the δ^2^H and δ^18^O values obtained from IAEA WISER (Water Isotope System for data analysis visualization and Electronic Retrieval database) [34]. The slope variation of each LMWL with the GMWL allows assessment of the alteration of climatic conditions in a geographical location and year [35,36]. In general, the LMWL for the Mediterranean countries studied displayed a depletion of slope in comparison with the GMWL, which is related to rise in temperature and reduction of precipitation [35]. The current climatic change and global warming scenario is dominated by the rising of mean temperature in the Mediterranean basin together with the reduction of the number of rainfall episodes and the amount of water [37,38]. A more detailed analysis of δ^2^H and δ^18^O by country and year shows that in 2016 all studied countries showed the same depletion slope, which may be correlated to a rise in drought conditions. However, in 2017, the trends were different. In the case of French samples in 2017, a slope parallel to the GMWL is displayed, while in 2016 its slope displays a conspicuous depletion. The average value of temperature and precipitation (Table S2) in France showed a significant (*p* < 0.05) depletion of 39% in rainfall in 2017. A possible explanation may be the existence of an irrigation process, since, in this year, the cultivation area suffered a significant reduction in rainfall (over 30%, Table S2). Irrigation may alter the natural δ^2^H and δ^18^O value [39,40]. Therefore, the non-alteration of the 2017 slope in France may be due to the anthropogenic factor. Displacement may be seen on the y-axis (δ^18^O composition), which may be related to the isotope irrigation water value. In the case of Portugal, slopes in different harvest years display the same value. However, in 2017, the LMWL showed a shift in δ^18^O composition, i.e., in the y-intercept. This may be supported by a significant reduction in rainfall of 31% (Table S2). On the other hand, Turkey shows a slight change in slope in 2017. The analysis of precipitation data (Table S2) showed a rise in rainfall of 21%, but this is not significant (*p* > 0.05). The good correlation of LMWL (correlation between treated δ^2^H and δ^18^O of VOOs), as well as their connection to the GMWL, conditioned by inherent geoclimatic factors (temperature, latitude, sea distance, rainfall, etc.), confirm the potential use of isotope composition of VOOs for their geoclimatic characterization. Some recent works have explored this possibility in VOO samples produced from the same olive variety and harvest year in different countries, for example, in Italy [19] and Spain [20].

### 3.3. Chemometric Analysis

**Figure 3:**
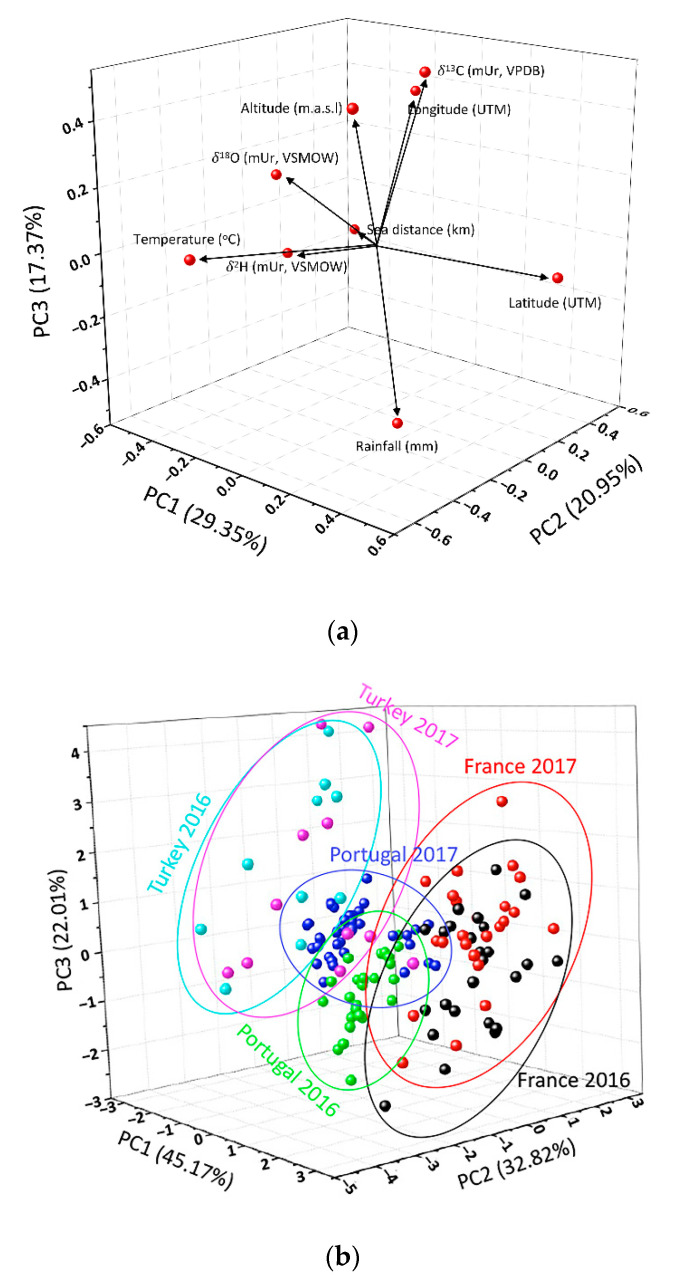
Principal components analysis (PCA). (**a**) 3D Plot of component weights for the geographical variables and the carbon, oxygen and hydrogen (*δ*^13^C, *δ*^18^O and *δ*^2^H) isotope composition of VOO samples; (**b**) 3D Score plot of VOO samples from France, Portugal and Turkey in 2016 and 2017.

Principal component analysis (PCA) was employed on the one hand to identify the possible correlation between independent (stable isotopes of VOO samples) and dependent (geoclimatic parameters of produced areas) variables (Figure 3a), and on the other hand to sort the VOO samples by geographical region and harvest year (Figure 3b). The analysis of the three first components (component 1: 29.35%, component 2: 20.95%, and component 3: 17.37%) can explain up to 67% of the total variance (Table S3). The scatterplot of the loadings of PC-1 vs. PC-2 vs. PC-3 (Figure 3a) showed that *δ*^2^H values were directly correlated with temperature, sea distance and altitude, and negatively with latitude, longitude and rainfall. In contrast, *δ*^18^O values were correlated with longitude, altitude and temperature, while longitude and rainfall were negatively correlated with oxygen isotope composition. These correlations were previously observed in other articles by the authors. For example, Jiménez-Morillo et al., [11], using for the first time the analytical pyrolysis–compound specific isotope analysis (Py-CSIA), found that temperature and sea distance were directly correlated with the hydrogen isotope composition of specific compounds of EVOO samples from five Mediterranean countries. However, other authors showed inversely correlation between *δ*^18^O and sea distance [12]. With respect to carbon isotope (*δ*^13^C), this displays direct correlation with longitude and altitude but correlates inversely with rainfall. Camin et al. [41] and Jiménez-Morillo et al. [12] observed the same correlations between carbon isotope composition of olive oil samples and the geoclimatic factors. More rain will cause plant’s stomata to stay open longer, causing more isotopically light CO_2_ molecules to pass inside the leaf and the *δ*^13^C value to drop, relative to plants growing in a more arid environment. The existing correlations observed in Figure 3a were corroborated by a multi linear regression (MLR) approach. The MLR, using the geoclimatic parameters of the VOO producer locals (*n* = 138) from the studied Mediterranean countries, led to significant (*p* < 0.05) forecasting models for the carbon, hydrogen and oxygen isotope composition for the harvest year.

**Figure 4:**
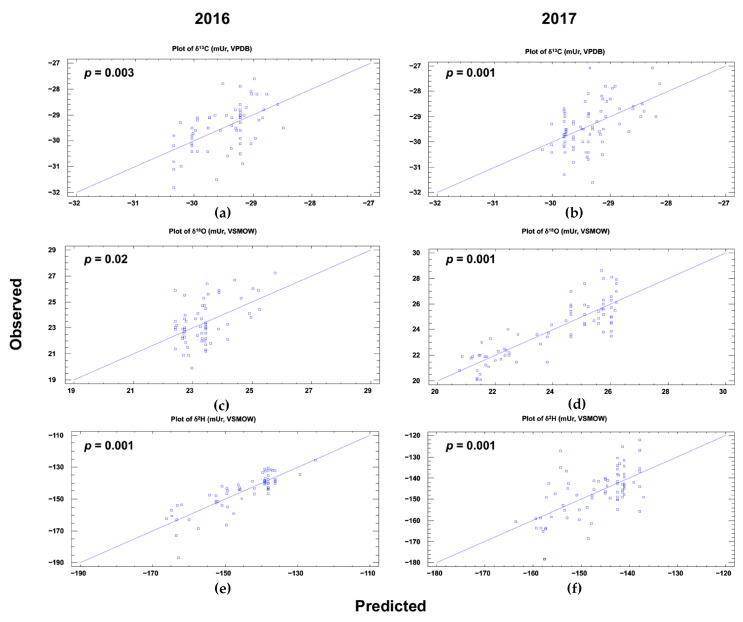
Observed vs. predicted values for carbon, oxygen and hydrogen isotopic composition in two harvest years (2016 and 2017) calculated by multiple linear regression (MLR) using geoclimatic parameters of VOO producer areas as predictors. (**a**,**b**) plots of δ^13^C from 2016 and 2017; (**c**,**d**) plots of δ^18^O from 2016 and 2017; (**e**,**f**) plots of δ^2^H from 2016 and 2017.

Figure 4 plots the observed vs. predicted values of each models for carbon, hydrogen and oxygen in 2016 and in 2017. The model validation was confirmed by a strict criterion based on comparing the MLR cross-validation tests (observed vs. predicted) with the alternative model computed from the fully randomized isotope values of VOO samples studied in this work (data no shown).

This cross-validation method has been previously used in several works [11,12,42]. The MLR analysis allowed us to obtain the Equations (1)–(6) of the prediction model, using automatic backwards variable selection:

δ^13^C (2016) = −42.24 + 0.24 × Latitude + 0.02 × Longitude − 0.01 × Rainfall + 0.22 × Temperature + 0.01 × Oceanic distance (1)

δ^13^C (2017) = −39.91 + 0.20 × Latitude + 0.02 × Longitude − 0.01 × Rainfall + 0.10 Temperature + 0.01 × Oceanic distance (2)

δ^18^O (2016) = 27.61 − 0.68 × Latitude + 0.08 × Longitude + 0.01 × Rainfall − 0.14 × Temperature + 0.02 × Oceanic distance (3)

δ^18^O (2017) = 55.68 − 0.67 × Latitude − 0.02 × Longitude − 0.01 × Rainfall − 0.28 × Temperature + 0.01 × Oceanic distance (4)

δ^2^H (2016) = −83.92 − 2.83 × Latitude + 0.03 × Longitude + 0.03 × Altitude + 0.05 × Rainfall+ 1.01 × Temperature + 0.02 × Oceanic distance (5)

*δ*^2^H (2017) = −234.01 + 0.70 × Latitude − 0.34 × Longitude + 0.03 × Altitude + 0.03 × Rainfall + 2.15 × Temperature + 0.04 × Oceanic distance (6)

In this way, the new predicting isotope value models (Equations (1)–(6)) will allow forecasting of changes that foodstuffs will undergo in different climate change scenarios, and adapting the criteria for the protection of designations of origin, safeguarding society from possible food fraud. In addition, they could be used to assess the isotopic composition of virgin olive oils under different climatic change scenarios (from the mildest to the most severe). However, it is known that the isotopic forecasting models developed should be corrected every year owing to intrinsic fluctuations in isotopic composition. Therefore, the next step will be the validation and correction of the possible isotopic alterations, generating correction factors.

On the other hand, the 3D scores in the PCA plot of VOO samples (Figure 3b), generated using their unique isotopic composition, displayed three clusters, one for each country. In the case of the French and Turkish cluster, their separation is dominated by the difference in the stable isotope value of hydrogen and oxygen. The French samples displayed higher isotope values than the VOO samples from Turkey (Figure 2b,c). The Portuguese cluster showed an intermediate location in the 3D PCA plot (Figure 3b). These samples show no significant difference with respect to the other two regional VOO samples (Figure 2b,c). Although the samples are well-grouped into three clusters depending on the country, a more detailed study shows that the samples can be separated slightly depending on the harvest year. The most prominent case is the samples from Portugal (Figure 3b). This difference may be directly related to the variation in the *δ*^18^O (Figure 2b), as well as the significant difference in rainfall (Table S2). With respect to the other Mediterranean VOO samples, this difference between cultivation years was not so clear.

**Figure 5:**
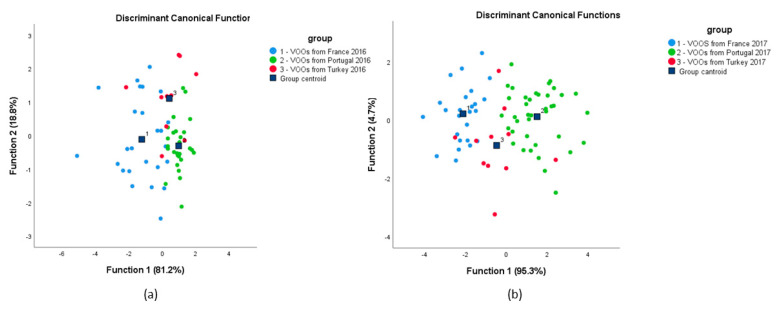
Linear discriminant analysis performed on isotope composition of EVOO samples (*δ*^13^C, *δ*^18^O and *δ*^2^H) from 2016 (**a**) and 2017 (**b**).

In order to verify if a better classification of olive oils can be achieved, a Linear Discriminant Analysis (LDA) was also performed. Figure 5 shows the scatter plot for olive oils from the three countries in each year. Two discriminant functions were constructed based on Wilks’ Lambda values, which explained 100% of the variance, 81.2% of the total variance explained by function 1 and 18.8% explained by function 2, for 2016 olive oils; and 95.3% of the total variance explained by function 1 and 4.7% explained by function 2, for 2017 olive oils (Table S5). The Wilks’ Lambda values were, for function 1 and 2, 0.377 and 0.795 with a *p*-value of 0.000 and 0.001, for 2016 olive oils; and 0.230 and 0.878 with a *p* value of 0.000 and 0.010, for 2017 olive oils, which implies that there are significant differences among the three regions and the first function has higher discrimination power, since Wilk’s Lambda is lower and *p*-value is equal to 0. Discriminant functions can be shown as:

Function 1 (2016) = 20.082 − 0.074 δ^13^C − 0.174 δ^18^O + 0.125 δ^2^H (7)

Function 2 (2016) = −0.196 + 0.434 δ^13^C + 0.593 δ^18^O − 0.006 δ^2^H (8)

Function 1 (2017) = −29.207 − 0.535 δ^13^C +0.733 δ^18^O + 0.028 δ^2^H (9)

Function 2 (2017) = 9.266 − 0.497 δ^13^C − 0.392 δ^18^O + 0.099 δ^2^H (10)

Table S5 summarizes the performance of the discriminant models regarding the classification of olive oil samples, and shows that the application of cross-validation (CV) exposes some misclassifications, mainly regarding samples from Turkey. For 2016 samples, 79.0% of the olive oil was correctly classified and this value diminished to 74.2% after cross validation; for 2017 samples, results were better, with 92.9% of the cases correctly classified before and after CV. Olive oils from Portugal performed better than the others with the highest percentage of correctly classified olive oils, according to country of origin.

## 4. Conclusions

In light of the results, the combination of multivariate isotopic analysis of VOO samples and chemometrics allows discrimination between geoclimatic regions but also among cultivation years. Therefore, it presents a powerful tool to fight against food fraud in the VOO sector. In addition, the design of this work has allowed the generation of isotopic forecasting models using the geoclimatic parameters as predictive factors. Therefore, the interpolation of geoclimatic data (mainly temperature and rainfall) under different climatic change scenarios (from benevolent to drastic) will allow us to assess the isotopic alteration of virgin olive oil in future scenarios. 

This displays a great potential for establishing new correction factors to determine the geographical origin (Designation of Origin) of olive oils.

## Supplementary Materials

The following are available online at https://www.mdpi.com/2304-8158/9/12/1855/s1, Table S1: Varieties, origin, region, geo-climatic information and stable isotope composition of VOO samples; Table S2: Average and Standard deviation of Temperature and Rainfall in France, Portugal and Turkey in two years (2016 and 2017). Alteration of rainfall amount (%); Table S3: Principal component loading scores; Table S4: Variable standardized canonical discriminant function coefficients along with discriminant functions classification parameters; Table S5: Overall classification performance (count and %) of the LDA model, with and without cross validation (CV), for the three countries differentiation study.
