# Supplementary material for: Combination of Stable Isotope Analysis and Chemometrics to Discriminate Geoclimatically and Temporally the Virgin Olive Oils from Three Mediterranean Countries
Source: Foods. 2020 Dec 12;9(12):1855. doi: 10.3390/foods9121855 (PMC7763173; doi:10.3390/foods9121855)
Supplement: Supplementary file 1 [file foods-09-01855-s001.pdf]

Table S1. Varieties, origin, region, geo-climatic information and stable isotope composition of EVOO samples.

Country and year	Sample code	Latitude (UTM)	Longitude (UTM)	Altitude (m a.s.l.)	Rainfall (mm)	Temperature (°C)	Sea distance (km)	δ13C (mUr, VPDB)	δ18O (mUr, VSMOW)	δ2H (mUr, VSMOW)
France 2016	AG001	43.55	5.16	50	515.2	15.62	23.09	-30.9	22.0	-162.3
	AG026	43.37	5.65	240	515.2	15.4	20.32	-29.0	22.6	-162.9
	AG032	43.89	5.14	150	515.2	15.62	55.77	-29.1	22.6	-160.7
	AG035	44.03	5.98	400	788.7	11.59	100.7	-29.5	21.8	-166.2
	AG037	43.84	5.79	450	515.2	13.9	75.65	-29.2	21.9	-168.4
	AG044	43.83	5.96	550	515.2	13.9	78.83	-28.8	23.2	-147.5
	CA007	44.46	4.40	240	953.5	13.85	111.1	-29.6	22.9	-144.4
	CA022	43.67	6.98	300	713.7	12.73	13.55	-29.7	20.9	-147.9
	CA051	43.68	6.97	350	713.7	12.73	14.77	-29.6	20.9	-153.8
	CA085	43.79	7.22	200	760	14.48	11.17	-30.4	19.9	-151.4
	OL052	43.41	3.17	60	902.6	18.1	22.05	-29.0	21.5	-149.8
	OL053	43.63	3.45	90	902.6	13.95	31.41	-29.3	22.0	-154.8
	OL067	43.26	3.26	20	902.6	18.1	4.24	-31.5	23.5	-143.9
	PI042	43.23	2.65	50	822.1	16.3	40.61	-29.0	23.7	-141.8
	PI048	43.63	3.45	90	902.6	14.0	31.41	-31.0	24.7	-144.9
	PI049	43.64	4.81	20	582.4	16.7	29.58	-30.1	22.2	-163.0
	PI072	43.72	4.42	80	582.4	16.5	29.69	-28.2	23.7	-153.7
	SA014	43.55	5.23	100	515.2	15.62	27.33	-28.7	24.1	-156.9
	SA015	43.72	4.84	40	582.4	16.7	40.99	-28.2	25.3	-153.8
	SA030	43.74	4.72	30	582.4	16.7	34.75	-27.6	24.7	-172.8
	SA031	43.73	4.74	50	582.4	16.7	35.16	-29.9	23.1	-186.7
	TA054	44.39	5.10	400	788.7	13	114.52	-28.8	26.4	-146.7
	TA055	44.37	5.13	400	788.7	13	117.07	-29.5	25.6	-142.9
	TA078	44.36	5.13	300	788.7	13	117.07	-29.4	25.9	-151.2
	TA080	44.36	5.14	300	788.7	13	117.07	-29.1	25.7	-152.5

Table S1. Continue

Country and year	Sample code	Latitude (UTM)	Longitude (UTM)	Altitude (m a.s.l.)	Rainfall (mm)	Temperature (°C)	Sea distance (km)	δ13C (mUr, VPDB)	δ18O (mUr, VSMOW)	δ2H (mUr, VSMOW)
France	AG210	43.37	5.65	240	367.4	15.84	19.84	-28.3	23.0	-152.9
2017	AG217	43.84	5.79	450	367.4	14.15	77.91	-29.0	21.7	-161.6
	AG219	43.83	5.96	550	367.4	14.15	81.21	-27.1	22.3	-141.1
	AG256	43.89	5.14	150	367.4	16	62.33	-27.8	23.3	-127.1
	AG261	44.03	5.98	400	587.5	11.85	97.79	-29.6	21.5	-168.4
	AG263	43.55	5.16	50	367.4	16	24.46	-29.5	21.2	-163.7
	CA231	43.79	7.22	200	559.3	14.7	11.26	-29.0	21.9	-155.2
	CA266	43.63	6.85	380	490.5	12.98	13.28	-27.9	21.9	-144.7
	CA267	43.72	7.15	230	559.3	14.7	6.75	-28.4	21.8	-142.5
	CA268	43.67	6.98	300	490.5	12.98	13.99	-27.8	21.1	-147.3
	OL220	43.41	3.17	60	575.3	18.19	21.01	-30.5	21.9	-153.9
	OL221	43.63	3.45	90	575.3	14.01	33.2	-30.4	21.6	-158.4
	OL273	43.26	3.26	20	575.3	18.19	2.77	-31.6	20.8	-147.9
	OL236	43.24	2.79	40	790.5	16.38	31.02	-29.7	20.1	-148.2
	PI242	43.82	4.02	90	320.6	16.75	28.72	-29.5	20.8	-142.4
	PI244	43.72	4.42	80	320.6	16.75	29.96	-28.2	20.5	-149.2
	PI277	43.24	2.79	40	790.5	16.38	31.02	-27.1	20.2	-149.6
	SA226	43.72	4.84	40	320.6	17.14	37.63	-30.0	20.1	-178.2
	SA228	43.73	4.74	50	320.6	17.14	34.93	-28.3	22.0	-163.6
	SA282	43.59	5.13	100	367.4	16	29.21	-29.6	21.9	-164.1
	SA286	43.74	4.72	30	320.6	17.14	34.86	-29.7	22.0	-165.2
	TA248	44.36	5.14	300	587.5	13.34	115.95	-28.6	22.2	-145.5
	TA249	44.36	5.13	300	587.5	13.34	115.95	-28.7	22.0	-141.4
	TA290	44.37	5.14	380	587.5	13.34	115.95	-28.5	22.4	-143.6
	TA291	44.37	5.10	400	587.5	13.34	115.95	-28.8	22.0	-149.4

Table S1. Continue

Country and year	Sample code	Latitude (UTM)	Longitude (UTM)	Altitude (m a.s.l.)	Rainfall (mm)	Temperature (ºC)	Sea distance (km)	δ¹³C (mUr, VPDB)	δ¹⁸O (mUr, VSMOW)	δ²H (mUr, VSMOW)
Portugal 2016	Cob 1	38.17	-7.72	163	679.3	18.01	92.76	-29.8	23.7	-138.5
	Cob 3	38.06	-8.11	140	738.1	18.17	61.99	-30.8	23.0	-137.5
	Cob 4	38.17	-7.72	163	679.3	18.01	92.76	-30.4	23.0	-138.9
	Cob 5	38.88	-7.16	328	679.3	17.6	197.1	-29.0	23.4	-142.3
	Cob 6	38.17	-7.72	163	679.3	18.01	92.76	-29.5	25.5	-131.3
	Cob 7	38.41	-7.72	190	679.3	18.01	96.27	-29.1	22.9	-136.9
	Cob 8	38.88	-7.16	328	679.3	17.6	197.1	-28.6	22.2	-143.8
	Cor 1	38.88	-7.16	328	679.3	17.6	197.1	-29.1	22.9	-143.0
	Cor 2	38.17	-7.72	163	679.3	18.01	92.76	-30.2	22.3	-140.0
	Cor 3	38.06	-8.11	140	738.1	18.17	61.99	-30.2	22.9	-139.2
	Cor 4	37.94	-7.60	218	625.3	18.41	114.17	-29.1	24.0	-138.9
	Gal 1	38.06	-8.11	140	738.1	18.17	61.99	-29.8	25.9	-132.0
	Gal 2	38.36	-7.29	186	679.3	17.99	132.69	-29.1	22.4	-133.3
	Gal 4	38.88	-7.16	328	679.3	17.6	197.1	-28.9	23.0	-135.1
	Verd 1	38.88	-7.16	328	679.3	17.6	197.1	-29.0	23.1	-140.0
	Bla 1	38.88	-7.16	328	679.3	17.6	197.1	-27.9	21.2	-132.3
	Mad 1	38.88	-7.16	328	679.3	17.6	197.1	-29.9	23.3	-137.5
	Arb 1	38.88	-7.16	328	679.3	17.6	197.1	-29.6	21.3	-139.8
	Arb 3	38.41	-7.72	190	679.3	18.01	96.27	-29.2	22.3	-132.4
	Arb 4	38.17	-7.72	163	679.3	18.01	92.76	-29.9	22.7	-138.2
	Arb 5	38.06	-8.11	140	738.1	18.17	61.99	-31.1	23.5	-138.2
	Car 1	38.88	-7.16	328	679.3	17.6	197.1	-30.1	24.5	-130.7
	Azei 1	38.88	-7.16	328	679.3	17.6	197.1	-29.3	22.6	-143.6
	Pic 1	38.88	-7.16	328	679.3	17.6	197.1	-30.5	21.7	-146.6
	Pic 3	38.41	-7.72	190	679.3	18.01	96.27	-30.4	22.2	-139.5
	Pic 4	38.17	-7.72	163	679.3	18.01	92.76	-30.1	22.9	-140.6
	Pic 5	38.06	-8.11	140	738.1	18.17	61.99	-31.8	21.4	-140.1

Table S1. Continue

Country and year	Sample code	Latitude (UTM)	Longitude (UTM)	Altitude (m a.s.l.)	Rainfall (mm)	Temperature (°C)	Sea distance (km)	$\delta^{13}C$ (mUr, VPDB)	$\delta^{18}O$ (mUr, VSMOW)	$\delta^{2}H$ (mUr, VSMOW)
Portugal 2017	Gal1	39.07	-7.46	308	378	16.95	160.35	-29.5	25.5	-133.1
	Gal4	38.08	-7.27	193	440.7	18.52	142.85	-30.4	27.6	-148.9
	Gal11	37.94	-7.44	229	475.9	18.48	127.7	-29.2	24.6	-147.7
	Gal13	38.55	-7.84	263	440.7	18.55	88.91	-29.5	25.9	-148.8
	Gal18	38.88	-7.16	322	440.7	18.18	153.94	-30.0	26.0	-155.6
	Gal21	40.38	-7.93	364	577.4	14.49	83.72	-29.3	25.8	-150.2
	Arb1	38.08	-7.27	193	440.7	18.52	142.85	-28.9	27.0	-150.2
	Arb2	37.94	-7.44	229	475.9	18.48	127.7	-29.8	23.5	-139.4
	Arb4	38.55	-7.84	263	440.7	18.55	88.91	-29.6	24.5	-146.5
	Arb7	38.88	-7.16	322	440.7	18.18	153.94	-29.0	23.8	-126.8
	Arb10	38.17	-7.76	157	440.7	18.55	97.36	-30.1	24.5	-140.8
	Arb11	38.06	-8.11	136	444.5	18.71	65.12	-30.3	25.8	-141.6
	Arb12	39.92	-8.15	396	505.8	16.47	84.49	-30.0	24.7	-142.0
	Cob1	38.08	-7.27	193	440.7	18.52	142.85	-29.0	27.9	-154.7
	Cob3	37.94	-7.44	229	475.9	18.48	127.7	-28.7	24.5	-144.2
	Cob5	38.88	-7.16	322	475.9	18.18	153.94	-28.9	25.6	-149.0
	Cob8	38.17	-7.76	157	440.7	18.55	97.36	-29.3	25.4	-146.4
	Cob9	38.38	-7.56	213	440.7	18.55	120.6	-29.7	28.6	-138.5
	Cob10	40.38	-7.93	364	577.4	14.49	83.72	-30.8	25.4	-135.7
	Cob11	40.38	-7.93	364	577.4	14.49	83.72	-29.9	25.9	-138.3
	Cob12	40.38	-7.93	364	577.4	14.49	83.72	-29.8	27.0	-142.8
	Cob13	40.38	-7.93	364	577.4	14.49	83.72	-30.2	23.6	-141.5
	Verd1	37.94	-7.44	229	475.9	18.48	127.7	-30.2	25.0	-138.0
	Verd3	37.94	-7.44	229	475.9	18.48	127.7	-29.7	26.3	-134.4
	Car1	37.94	-7.44	229	475.9	18.48	127.7	-31.3	25.7	-145.1
	Car3	38.54	-7.84	263	440.7	18.55	88.91	-29.5	24.4	-145.4

Table S1. Continue

Country and year	Sample code	Latitude (UTM)	Longitude (UTM)	Altitude (m a.s.l.)	Rainfall (mm)	Temperature (ºC)	Sea distance (km)	$\delta^{13}C$ (mUr, VPDB)	$\delta^{18}O$ (mUr, VSMOW)	$\delta^{2}H$ (mUr, VSMOW)
Portugal 2017	Cor1	38.08	-7.27	193	440.7	18.52	142.85	-30.0	26.1	-130.6
	Cor3	37.94	-7.44	229	475.9	18.48	127.7	-28.8	23.9	-142.8
	Cor5	38.54	-7.84	263	440.7	18.55	88.91	-29.7	27.6	-125.3
	Cor6	37.94	-7.44	229	475.9	18.48	127.7	-29.8	25.0	-143.4
	Cor9	40.38	-7.93	364	577.4	14.49	83.72	-30.3	23.4	-146.5
	Pic1	38.54	-7.84	263	440.7	18.55	88.91	-29.6	25.2	-143.0
	Pic3	38.88	-7.16	322	440.7	18.18	153.94	-28.1	25.2	-144.1
	Pic6	38.17	-7.76	157	440.7	18.55	97.36	-30.4	24.9	-147.0
	Mad1	38.88	-7.16	322	440.7	18.18	153.94	-30.4	25.6	-136.9
	Bla1	37.94	-7.44	229	475.9	18.48	127.7	-30.1	26.6	-131.6
	Bla4	38.88	-7.16	322	440.7	18.18	153.94	-30.7	28.0	-135.4
	Bla7	40.38	-7.93	364	577.4	14.49	83.72	-29.2	25.2	-134.1
Turkey 2016	AYV1	39.32	26.70	10	637.1	18.24	5.86	-27.8	24.1	-141.0
	MEM1	38.45	27.22	50	574.1	18.31	35.74	-30.3	25.9	-142.7
	TVY1	36.27	32.30	20	724.7	22.2	1.88	-30.6	23.8	-134.6
	BEY1	36.78	31.45	9	807.1	24.31	2.84	-28.1	25.3	-125.6
	DMT1	37.62	27.43	50	470.1	19	24.6	-29.6	26.0	-159.0
	HAL1	36.10	34.20	416	262.3	18.04	1	-28.8	23.3	-143.0
	SUL1	36.92	34.85	23	486.3	21.88	13.5	-28.2	24.4	-138.8
	HSB1	36.10	34.20	416	262.3	18.04	1	-29.1	22.1	-147.1
	NZP1	37.02	37.77	561	397.6	16.88	143.89	-29.5	27.2	-131.5
	KLS1	36.70	37.10	709	262.3	17.01	79.54	-28.6	26.7	-132.3
Turkey 2017	AYV2	39.32	26.70	10	759.7	17.79	5.86	-28.4	23.6	-158.9
	MEM2	38.45	27.22	50	632.5	17.97	35.74	-30.6	23.7	-159.3
	TVY2	36.27	32.30	20	738.5	21.76	1.88	-29.5	22.9	-135.1
	BEY2	36.78	31.45	9	915.2	23.91	2.84	-28.8	24.0	-140.1

Table S1. Continue

Country and year	Sample code	Latitude (UTM)	Longitude (UTM)	Altitude (m a.s.l.)	Rainfall (mm)	Temperature (ºC)	Sea distance (km)	δ13C (mUr, VPDB)	δ18O (mUr, VSMOW)	δ2H (mUr, VSMOW)
	GEM2	40.72	29.25	5	718	15.96	1	-29.4	22.5	-160.7
	DMT2	37.62	27.43	50	693.4	18.68	24.6	-29.4	24.4	-178.1
	HAL2	36.10	34.20	416	483.5	20.6	1	-28.7	21.5	-159.7
	SUL2	36.92	34.85	23	566.8	21.75	13.5	-29.2	23.6	-163.6
	HSB2	36.10	34.20	416	483.5	20.6	1	-29.3	23.4	-154.9
	NZP2	37.02	37.77	561	278	17.21	143.89	-27.7	28.1	-136.5
	KLS2	36.70	37.10	709	204	16.91	79.54	-29.0	24.7	-158.7

AG – Aglandau; CA – Cailletier; OL – Olivière; PI – Picholine; SA – Salonenque; TA – Tanche; Cob – Cobrançosa; Cor – Cordovil de Serpa; Gal – Galega Vulgar; Verd – Verdeal Alentejana; Bla – Blanqueta; Mad – Madural; Arb – Arbequina; Car – Carrasquenha, Azei – Azeiteira; Pic – Picual; AYV – Ayvalik; MEM – Memecik; TVY – Tavsan Yuregi; BEY – Beilik; GEM – Gemlik; DMT – Domat; HAL – Halhali; SUL – Sariulak; HSB – Hasebi; NZP – Nizip Yaglik; KLS – Killis Yaglik.

Table S2. Average and Standard deviation of Temperature and Rainfall in France, Portugal and Turkey in two years (2016 and 2017). Alteration of rainfall amount (%).

Country	Year	Temperature (ºC)	Rainfall (mm)	Rainfall (%) alteration
France	2016	14.8±1.8	700.8±152.8[a]	30.64*
	2017	15.2±1.8	486.1±143.3[b]	
Portugal	2016	17.9±0.3	688.2±26.4[a]	31.06*
	2017	17.6±1.6	474.4±53.1[b]	
Turkey	2016	19.4±2.5	488.4±196.9[a]	-20.49
	2017	19.4±2.5	588.5±213.3[a]	

One-way ANOVA. The different letters indicate significant ($P < 0.05$) differences between years according to the Tukey test.

* Significant alteration of the rainfall amount (P <0.05)

Table S3. Principal component loading scores

Principal Components	Eigenvalue	Percentage of variance	Cumulative percentage
1	2.64	29.35	29.35
2	1.89	20.95	50.31
3	1.56	17.37	67.68
4	0.91	10.06	77.74
5	0.69	7.65	85.39
6	0.60	6.66	92.05
7	0.48	5.27	97.32
8	0.16	1.82	99.14
9	0.08	0.86	100

Table S4. Variable standardized canonical discriminant function coefficients along with discriminant functions classification parameters.

Variables	VOOs from 2016		VOOs from 2017	
	Function		Function	
	1	2	1	2
δ^2H	0.965*	0.260	0.330	0.768*
$\delta^{18}O$	-0.004	0.927*	0.889*	-0.347
$\delta^{13}C$	-0.113	0.485*	-0.259*	-0.227
Eigenvalue	1.108	0.257	2.821	0.139
% of Variance	81.2	18.8	95.3	4.7
Cumulative %	81.2	100.0	95.3	100.0
Canonical Correlation	0.725	0.452	0.859	0.350

* Largest absolute correlation between each variable and any discriminant function

Table S5. Overall classification performance (count and %) of the LDA model, with and without cross validation (CV), for the three countries differentiation study.

**Original class**	**Predicted class (LDA model)**					
	**France**		**Portugal**		**Turkey**	
	**Without CV**	**With CV**	**Without CV**	**With CV**	**Without CV**	**With CV**
France (2016)	17 (68.0%)	16 (64.0%)	6 (24.0%)	7 (28.0%)	2 (8.0%)	2 (8.0%)
France (2017)	25 (100.0%)	25 (100.0%)	0 (0.0%)	0 (0.0%)	0 (0.0%)	0 (0.0%)
Portugal (2016)	0 (0.0%)	0 (0.0%)	26 (96.3%)	25 (92.6%)	1 (3.7%)	2 (7.4%)
Portugal (2017)	0 (0.0%)	0 (0.0%)	39 (100.0%)	39 (100.0%)	0 (0.0%)	0 (0.0%)
Turkey (2016)	1 (10.0%)	1 (10.0%)	3 (30.0%)	4 (40.0%)	6 (60.0%)	5 (50.0%)
Turkey (2017)	2 (18.2%)	2 (18.2%)	4 (36.4%)	4 (36.4%)	5 (45.5%)	5 (45.5%)
